# The effectiveness of prescription drug monitoring programs at reducing opioid-related harms and consequences: a systematic review

**DOI:** 10.1186/s12913-019-4642-8

**Published:** 2019-11-01

**Authors:** Emily Rhodes, Maria Wilson, Alysia Robinson, Jill A. Hayden, Mark Asbridge

**Affiliations:** 1 Department of Community Health & Epidemiology, Dalhousie University, Halifax, NS Canada; 2 Department of Emergency Medicine, Dalhousie University, Halifax, NS Canada

**Keywords:** Prescription drug monitoring programs, Opioids, Harms, Consequences

## Abstract

**Background:**

In order to address the opioid crisis in North America, many regions have adopted preventative strategies, such as prescription drug monitoring programs (PDMPs). PDMPs aim to increase patient safety by certifying that opioids are prescribed in appropriate quantities. We aimed to synthesize the literature on changes in opioid-related harms and consequences, an important measure of PDMP effectiveness.

**Methods:**

We completed a systematic review. We conducted a narrative synthesis of opioid-related harms and consequences from PDMP implementation. Outcomes were grouped into categories by theme: opioid dependence, opioid-related care outcomes, opioid-related adverse events, and opioid-related legal and crime outcomes.

**Results:**

We included a total of 22 studies (49 PDMPs) in our review. Two studies reported on illicit and problematic use but found no significant associations with PDMP status. Eight studies examined the association between PDMP status and opioid-related care outcomes, of which two found that treatment admissions for prescriptions opioids were lower in states with PDMP programs (*p* < 0.05). Of the thirteen studies that reported on opioid-related adverse events, two found significant (*p* < 0.001 and p < 0.05) but conflicting results with one finding a decrease in opioid-related overdose deaths after PDMP implementation and the other an increase. Lastly, two studies found no statistically significant association between PDMP status and opioid-related legal and crime outcomes (crime rates, identification of potential dealers, and diversion).

**Conclusion:**

Our study found limited evidence to support overall associations between PDMPs and reductions in opioid-related consequences. However, this should not detract from the value of PDMPs’ larger role of improving opioid prescribing.

## Background

The misuse of opioids has reached epidemic levels across North America [1]. The crisis has been perpetuated, in part, by the over and inappropriate prescribing of opioids by health professionals, brought on by improvements in the treatment of chronic pain and pharmaceutical companies’ push to use opioids as a first line therapy [2, 3]. In the United States, 2.4 million people have a severe opioid use disorder (OUD), which involves dependence on opioid analgesic medications, heroin, or both [1, 4]. The increasing use of opioids has led to many consequences such as more frequent incidents of reported opioid misuse, drug diversion, crime, overdoses, and death [5–11].

In order to address the opioid crisis many regions have adopted preventative initiatives, including physician mentoring, continuing medical education on pain management, naloxone kits, and, the focus of the current study, prescription drug monitoring programs (PDMPs) [12–17]. PDMPs facilitate controlled substances like opioids to be prescribed in appropriate quantities, following best practice guidelines, not co-prescribed with potentially harmful substances, and only provided to patients when safe and necessary [14]. This is achieved by monitoring the prescribing practices of healthcare providers and identifying any patterns of drugs received by patients. Most PDMPs give healthcare providers the option to check PDMP data (patient profiles) before prescribing or dispensing opioids to a patient, allowing for more informed decision making. Broadly, PDMPs aim to restrict drug diversion and reduce opioid misuse-related harms [18, 19].

As of 2018, 49 states, the District of Columbia, and two U.S. territories (Guam & Puerto Rico) had implemented a PDMP [20]. A 2009 report estimated that start-up costs of a PDMP in the United States ranged between $450,000 and $1.5 million, with an average annual cost of $500,000 for maintaining a PDMP [21]. Significant resources are directed to these programs on an ongoing basis, and, as such, there is a need to evaluate their effectiveness.

An important measure of PDMP effectiveness is a reduction in opioid-related harms and consequences [22]. A 2018 scoping review addressed the association of PDMPs with fatal and non-fatal overdoses for any drugs [23]. This review found uncertainty in the evidence around an increase or decrease in fatal and nonfatal overdoes after the implementation of PDMPs [23]. However, individual studies on PDMP effectiveness report a broad range of other opioid-related outcomes, including dependence, emergency department (ED) visits, crime, treatment admissions, and illicit opioid use [24–28].

To date, no systematic review has been undertaken to synthesize the evidence on the impact of PDMPs on a range of opioid-related outcomes of interest to health professionals, decision-makers and other knowledge users, including associated harms and consequences. Understanding whether these programs work as intended is a crucial piece of information to combat the current opioid crisis.

## Methods

### Eligibility criteria

We included full published reports in all languages. Study designs were restricted to those that could draw conclusions on the effectiveness of PDMPs in reducing opioid-related consequences and harms (pre-post studies, controlled before/after, case-control, or cluster RCT designs). Two reviewers independently selected relevant studies from titles and abstracts. Any conflicts at the title and abstract level were discussed between the two reviewers. If consensus could not be achieved after discussion, the study was carried forward to the full-text screening level. Any conflicts at the full-text screening level were resolved by consulting with a third reviewer. We used Covidence software for all study screening [29]. Covidence, an online systematic review management platform, is a key element of Cochrane’s review production toolkit that facilitates study screening and data extraction.

We included studies of any jurisdiction (regional, provincial/state, national) or setting (clinic, hospital, system) where a PDMP had been implemented and where either a within jurisdiction comparison was made (pre- post- PDMP implementation) or a between jurisdiction comparison was made between those with and without a PDMP. We did not restrict studies by geographic region.

### Reporting the intervention

We considered the intervention of interest the presence of a PDMP, defined as a program that specifically monitors the outpatient prescription dispensing of opioids (or other drugs) by healthcare providers. To ensure broad scope of our review, we included all types of PDMPs.

### Outcomes of interest

We included opioid-related outcomes only in this review. It is important to note that we did not limit these outcomes to those related to prescription opioid use. We also included outcomes related to illicit opioids such as heroin, as the literature suggests that there is the potential for PDMPs to push people who use prescription opioids to illicit sources [6]. We did not include outcomes that addressed non-opioid analgesics, and other controlled substances monitored by PDMPs (e.g. benzodiazepines).

Opioid-related consequences and harms were grouped into categories by theme: opioid dependence (i.e. substance use disorders), opioid-related care outcomes (i.e. hospital visits, treatment program admissions), opioid-related adverse events (i.e. overdose, death), and opioid-related legal and criminal outcomes (i.e. arrests, diversion). Use and consequence outcomes could be linked to all opioids, or to specific types of opioids.

### Search strategy

We followed a standard systematic review approach, employing a predefined protocol, and structured the report according to PRISMA (Preferred Reporting Items for Systematic reviews and Meta-Analyses) guidelines [30].

To identify relevant publications, we established a uniform strategy for searching MEDLINE, Embase, CINAHL, PsycInfo, Web of Science, and grey literature including Dissertation and Theses Databases, CADTH, Health Canada, CIHI, and CMA Infobase, following the guidance of a medical librarian [31]. Furthermore, we manually searched reference lists of all included studies, related systematic reviews, and all additional relevant reviews identified in the electronic search. We also contacted authors of key publications and identified relevant conference abstracts, and reviewed personal libraries of the research team [32–35]. We systematically searched terms relevant to PDMPs, matching terms against possible subject headings (e.g. MeSH) and keywords. We performed the search on January 22, 2018 and included all pertinent publications published prior to that date (see Additional file 1).

### Risk of bias assessment

We assessed potential risk of bias for each study meeting selection criteria using the Quality of Prognostic Studies (QUIPS) tool. The QUIPS tool assesses risk of bias across six domains: study participation, study attrition, prognostic factor measurement, outcome measurement, study confounding, statistical analysis and reporting [36]. Specifically, we considered low risk of bias for the study sample if the response rate was > = 70% participation, moderate for 60–69.9%, and high for < 60%. Further, we considered whether studies adjusted for the following potential confounders: (a) The characteristics/features of PDMP (i.e. mandatory use), (b) demographic characteristics of sample (either individual, physician or jurisdiction level), and (c) presence of other related interventions in the study period (or other trends in substance use). Studies adjusting for at least two confounders were considered to have a low risk of bias on study confounding, 1 would be moderate, and 0 would be high.

### Data extraction, synthesis, and analysis

For all included studies, data extraction was completed by two independent reviewers using pre-tested data extraction forms developed in Covidence [29]. Any discrepancies in data extraction were discussed and the assessment of a third reviewer was sought for resolution. We extracted relevant study details (i.e. authors, year, jurisdiction, study design, sample size), population characteristics (i.e. providers, patients), interventions (i.e. included PDMP characteristics), and data sources (i.e. administrative, survey). Outcomes extracted included any unadjusted and adjusted associations between the presence of a PDMP, or the change in PDMP states and non-PDMP states over time, and each opioid-related harm or consequence outcome, as well as all variables controlled for.

We synthesized our data narratively. If studies had overlapping datasets, years of data, and jurisdictions, the study with most years of data for a dataset and jurisdiction was designated as the primary study in our narrative synthesis and the remaining studies were secondary. If a study presented both unadjusted and adjusted data, both were extracted; however, the adjusted data was included in our primary synthesis.

We used Excel 2016 for data management and Stata 15 for descriptive analyses and calculating pooled estimates [37, 38].

## Results

**Fig. 1:**
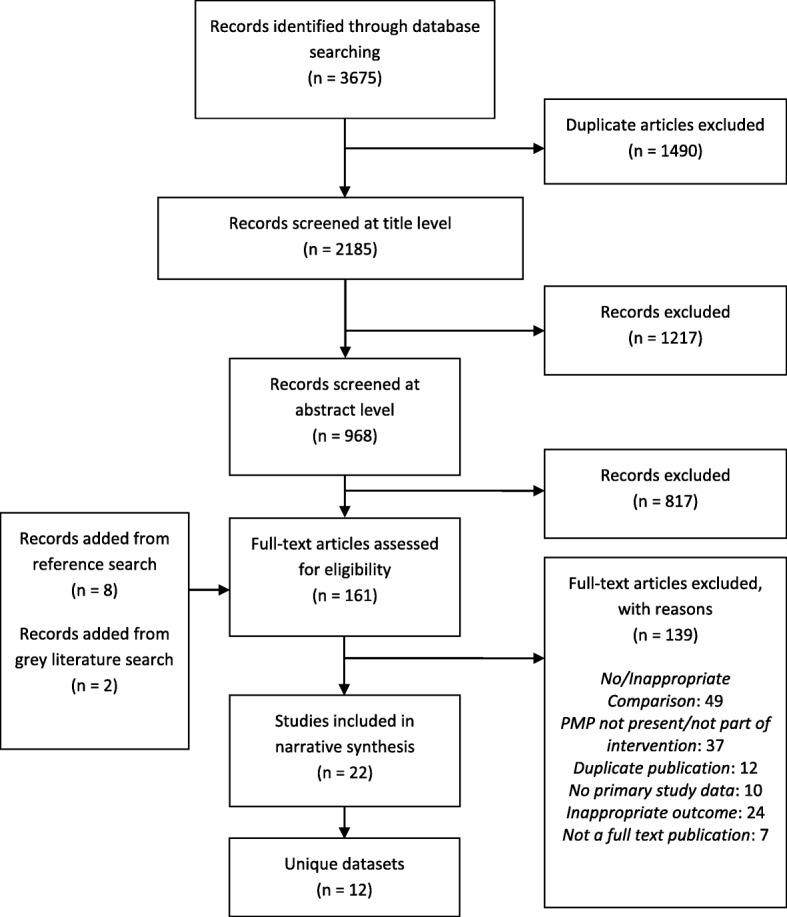
PRISMA flow chart depicting the study selection and inclusion process and results

The study selection process for this review is summarized in the PRISMA flow chart presented in Fig. 1. A total of 161 articles were assessed at the full text level, resulting in the inclusion of 22 studies addressing the association of PDMP status with opioid-related consequences or harms. All included studies took place in the United States and 72.7% presented findings among the general population (Table 1). Studies were published between 2006 and 2018 and include data years from 1992 to 2014. Twelve datasets were used, with the Treatment Episode Data Set (TEDS) being the most common, appearing in six publications with treatment admission outcomes. Studies with overlapping data differed in the years of data covered. Opioid-related consequences and harms are described in detail below and separated into the following four categories: illicit and problematic opioid use, opioid-related care outcomes, opioid-related adverse outcomes, and opioid-related legal and criminal outcomes.

**Table 1:** Study level characteristics of 22 included studies

Study ID	Included Jurisdiction with a PMP	Study Population	Study Design	Outcome Groups	Outcome Data Source, Years
Ali 2017 [24]	All states except MO and DC	general population	Pooled Cross-sectional Logit and generalized linear models	illicit opioid use^a^, opioid dependence^a^	National Survey on Drug Use and Health (NSDUH), 2004–2014
Birk 2017 [43] ^b^	All states that established PMPs between 1998 and 2012	general population, opioid-related deaths	Controlled interrupted time series	opioid-related care outcomes, opioid-related adverse events	Treatment Episode Data Set (TEDS), 1998–2012; National Vital Statistics System (NVSS), 1999–2012
Branham 2018 [27] ^b^	AL, AZ, CO, CT, IN, IA, LA, ME, MN, MS, NV, NM, NC, ND, OH, SC, TN, UT, VT, VA, WV, WY	people who use drugs admitted to treatment	Controlled interrupted time series	opioid-related care outcomes^a^	TEDS, 1992–2012
Dave 2017 [44] ^b^	AK, AZ, AR, CO, CT, DE, FL, GA, IA, KS, LA, ME, MD, MN, MS, MT, NE, NH, NJ, NM, NC, ND, OH, OR, SC, SD, TN, WA, WI, WY, VT, VA	general population	Controlled interrupted time series	opioid-related care outcomes	TEDS, 2003–2014
Delcher 2015 [50]^c^	FL	drug-related deaths	Controlled interrupted time series	opioid-related adverse events^a^	Florida Medical Examiners Commission, 2003–2012
Kim 2013 [51]	AL, AZ, CA, CO, CT, IL, IN, LA, ME, MI, MS, NM, NC, ND, OH, OK, SC, TN, VA, WV, NY, HI, ID, OA, RI, TX, MA, NV, UT, WY, KY	general population	Interrupted time series	opioid-related adverse events	CDC Wide-Ranging Online Data for Epidemiologic Research (WONDER); 1999–2008
Kinsell 2015 [40] ^c^	NY, FL	general population	Controlled before and after/ Difference-in-difference approach	opioid-related adverse events^a^, opioid-related care outcomes^a^	CDC WONDER & Florida Medical Examiner Drug Related Death Data & Florida Agency for Health Care Administration & New York State Inpatient Databases (SID)/ State Emergency Department Databases (SEDD), 2009–2012
Li 2014 [52]	MI, VA, NY, ME, MS, NC, CA, MA, TX, AZ, SC, TN, IL, RI, CO, CT, OH, AL, LA, OK, HI, ID, NM, KY, IN, UT, PA, WV, WY, ND, NV	general population	Controlled interrupted time series	opioid-related adverse events	NVSS, 1999–2008
Mallatt 2017 [55] ^c^	NSDUH: 49 states (excluding MO) ARCOS: AK, AL, AR, AZ, CA, CO, CT, DE, FL, GA, HI, IA, ID, IL, IN, KS, KY, LA, MA, MD, ME, MI, MN, MS, MT, NC, ND, NE, NH, NJ, NM, NV, NY, OH, OK, OR, PA, RI, SC, SD, TN, TX, UT, VA, VT, WA, WI, West VA, WY NIBRS: 26 states	general population	Repeated cross-sectional (NSDUH); interrupted time series (NIBRS & ARCOS)	opioid-related legal and criminal outcomes^a^	National Incident-Based Reporting System (NIBRS), 2004–2014
Maughan 2015 [25] ^c^	Detroit, Phoenix, San Francisco, Denver, Chicago, Boston, Miami, Minneapolis, New York City	general population	Generalized Estimate Equations with repeated measures	opioid-related care outcomes	eDrug Abuse Warning Network (DAWN), 2004–2011
McLaughlin 2016 [28]	AL, AZ, CA, CO, CT, IL, IN, IA, LA, ME, MI, MN, MS, NE, NM, NC, ND, OH, OK, SC, TN, VT, VA, WV, AK, NY, HI, ID, PA, RI, TX, MA, NJ, DE, NV, OR, UT, WA, WY, KS, SD, WI, FL, KY, MT	general population	Repeated cross sectional	illicit opioid use^a^	NSDUH, 2004–2012
Meara 2016 [48]	AL, AZ, CA, CO, CT, IL, IN, IA, LA, ME, MI, MN, MS, NE, NM, NC, ND, OH, OK, SC, TN, VT, VA, WV, AK, NY, HI, ID, PA, RI, TX, MA, NJ, DE, NV, OR, UT, WA, WY, WY, SD, WI, FL, KY, MT	insured population	Cohort; before and after	opioid-related adverse events	Medicare Provider Analysis and Review (MEDPAR), Outpatient, Carrier, and Medicare Beneficiary Summary files, 2006–2012
Meinhofer 2017 [53]	AL, AK, AZ, AR, CO, CT, DE, FL, GA, IA, WY, LA, ME, MD, MN, MS, MT, NE, NJ, NM, NC, ND, OH, OR, SC, SD, TN, VT, VA, WA, WI WY	general population	Interrupted time series	opioid-related adverse events^a^	NVSS, 2000–2013
Nam 2017 [45] ^c^	AL, AZ, CO, CT, IA, LA, ME, MN, MS, NC, ND, NM, OH, SC, TN, VA, VT, WV, WY	general population	Interrupted time series	opioid-related adverse events^a^	CDC WONDER, 1999–2014
Patrick 2016 [47] ^b^	WA, OR, AZ, NM, CO, WY, MT, SD, NE, WY, MN, IA, AR, LA, MS, AL, GA, FL, SC, NC, TN, VA, WV, OH, MD, DE, NJ, CT, VT, ME, AK, ND, WI, KY	general population	Controlled interrupted time series	opioid-related adverse events	CDC WONDER, 1999–2013
Paulozzi 2011 [46]	CA, HI, ID, IL, IN, KY, ME, MA, MI, NV, NM, NY, OK, PA, TX, UT, VA, WV, WY	general population	Controlled interrupted time series	opioid-related adverse events	CDC WONDER, 1999–2005
Pauly 2018 [49]	48 states (all but MO and PA), and DC	insured population	Interrupted time series	opioid-related adverse events	Truven Health Marketscan claims database, 2004–2014
Radakrishnan 2015 [39] ^b^	All that fall into 1992–2011	general population	Interrupted time series/ Repeated cross section	opioid-related care outcomes^a^, opioid-related adverse events^a^	TEDS, 1992–2010; NVSS, 1999–2010
Reifler 2012 [42] ^c^	CT, MA, ME, NY, PA, RI, AZ, CA, CO, HI, ID, NV, UT, WY, IA, IL, IN, MI, ND, OH, AL, KY, MS, NC, OK, SC, TN, TX, VA, WV, WA	general population	Controlled interrupted time series	opioid-related adverse events, opioid-related care outcomes^a^	Researched Abuse Diversion and Addiction-Related Surveillance System (RADARS), 2003–2009
Reisman 2009 [19]	CA, HI, IL, IN, MA, MI, NY, OK, TX, ID, KY, NV, RI, UT	people who use drugs admitted to treatment	Cross-sectional (with interrupted time series)	opioid-related care outcomes	TEDS, 2003
Simeone 2006 [41]	20 states	general population	Prospective cohort study	opioid-related care outcomes	TEDS, 1997 & 2003
Surratt 2014 [26] ^c^	FL	general population	Interrupted time series	opioid-related legal and criminal outcomes	RADARS, 2009–2012

^a^indicated outcome is available for heroin

^b^ study only includes states with PMPs implemented during the study period

^c^ study only focuses on some PMP states or jurisdictions within a country by choice, due to analytic constrictions, or due to availability of data

*AL* Alabama, *AK* Alaska, *AZ* Arizona, *AR* Arkansas, *CA* California, *CO* Colorado, *CT* Connecticut, *DE* Delaware, *FL* Florida, *GA* Georgia, *HI* Hawaii, *ID* Idaho, *IL* Illinois, *IN* Indiana, *IA* Iowa, *KS* Kansas, *KY* Kentucky, *LA* Louisiana, *ME* Maine, *MD* Maryland, *MA* Massachusetts, *MI* Michigan, *MN* Minnesota, *MS* Mississippi, *MO* Missouri, *MT* Montana, *NE* Nebraska, *NV* Nevada, *NH* New Hampshire, *NJ* New Jersey, *NM* New Mexico, *NY* New York, *NC* North Carolina, *ND* North Dakota, *OH* Ohio, *OK* Oklahoma, *OR* Oregon, *PA* Pennsylvania, *RI* Rhode Island, *SC* South Carolina, *SD* South Dakota, *TN* Tennessee, *TX* Texas, *UT* Utah, *VT* Vermont, *VA* Virginia, *WA* Washington, *WV* West Virginia, *WI* Wisconsin, *WY* Wyoming, *DC* District of Columbia

### Illicit and problematic opioid use

Two studies reported an association between PDMP status and heroin use, both using the same dataset and presenting adjusted models [24, 28]. These studies drew on multiple years of cross-sectional survey data, using an interrupted time series analysis that captured data from 36 states from 2004 to 2014 [24]. Neither study found any significant associations between PDMP status and heroin use. One study also examined the association between past-year opioid dependence and PDMP status; no significant association was observed [24]. See Additional file 2: Table S1 for individual study details.

### Opioid-related care outcomes

Eight studies examined the association between PDMP status and opioid-related care outcomes [19, 25, 27, 39–44]. One study, using CDC WONDER, SID, and SEDD data sets, reported on inpatient discharges in two jurisdictions with PDMPs from 2009 to 2012 using a difference-in-difference approach and found no change in the rate of discharges related to prescription opioids, and a slight increase in discharges related to heroin (β = 0.014, 90% CI [0.001–0.027]) post PDMP implementation in adjusted models [40]. That same study, along with an interrupted time series study on nine states from 2004 to 2011 using the DAWN data set, found no statistically significant associations when examining emergency department visits for all prescription opioids, Schedule II opioids, and heroin over time when comparing PDMP jurisdictions to non PDMP jurisdictions [25, 40].

Seven studies described opioid-related treatment admissions. Six of the seven used the TEDS dataset, while Reifler et al. used the RADARS dataset [19, 27, 39, 41–44]. Branham et al. covered the most years of data from TEDS; they found no association when examining the association between treatment admissions for heroin and PDMP status [27]. Branham et al. and Reifler et al. found no association between PDMP status and prescription opioid treatment admissions [27, 42]. In Branham et al.’s secondary analysis, they examined each of the states that implemented a PDMP during the study period (1992–2012) separately. They found that 13 out of 22 states saw a significant change in the average heroin admissions post PDMP – 10 states saw more admissions and three saw fewer. Furthermore, 11 US states reported a significant increase in average prescription opioid treatment admissions post-PDMP implementation [27]. See Additional file 2: Table S2 for individual study details.

### Opioid-related adverse events

Thirteen studies reported on opioid-related adverse events [39, 40, 42, 43, 45–53]. Of those studies, 10 reported on fatal opioid overdoses, with overlapping datasets in multiple studies [39, 40, 43, 45–47, 50, 51, 53, 54]. Four studies reported on heroin-related overdose deaths, none of which found any association with PDMP status in adjusted models [39, 40, 50, 53].

Six studies reported on overdose deaths related to both prescription and non-prescription opioids from two unique data sources – CDC WONDER and NVSS [39, 43, 45, 47, 50–52]. Both primary studies (with the most years of data available) reported no significant associations between opioid-related deaths and PDMP status [43, 45].

Five studies reported on fatal prescription opioid overdoses using three unique data sources – CDC WONDER, NVSS and state-specific inpatient and emergency databases [40, 45–47, 53]. No significant association between PDMP status and fatal prescription opioid overdose deaths was observed in adjusted models in any of these studies [40, 45, 46, 53].

Two studies reported on the association between specific opioid-related deaths and PDMP status [45, 50]. Nam et al. performed an interrupted time series analysis on data from 19 states that implemented PDMPs from 1999 to 2014 and found no association between methadone-related overdoses and PDMP status over time [45]. Delcher et al. performed a controlled interrupted time series analysis from 2003 to 2012 and observed a significant decline in oxycodone-caused overdoses in Florida post-PDMP implementation (*p* = 0.0079), but not in non-oxycodone related overdoses [50].

Two unique studies examined the association between non-fatal overdose and PDMPs [48, 49]. A study examining cohorts of Medicare beneficiaries for each year from 2006 to 2012 in 45 states found no association between PDMP status and the proportion of insurance beneficiaries experiencing non-fatal prescription opioid overdoses in adjusted models [48]. The second study was an interrupted time series of 49 state PDMPs from 2004 to 2014 and found that at baseline (2004), prescription opioid poisoning rates were higher in PDMP states than non PDMP states; however, the rate of prescription opioid poisonings over time decreased more quickly in PDMP states than in non PDMP states (β = − 0.005, 95% CI [− 0.008- -0.003]) [49]. Similarly, a separate study reported on intentional opioid poisonings for five drugs combined (fentanyl, hydromorphone, methadone, morphine, and oxycodone) and observed that, while rates were higher in PDMP states at baseline, rates reduced at a greater rate per quarter for PDMP states compared to non-PDMP states [42]. See Additional file 2: Table S3 for individual study details.

### Opioid-related legal and criminal outcomes

Two unique studies reported on three types of opioid-related criminal outcomes: crime rates, identification of potential dealers, and opioid diversion [26, 55]. Employing standardized, adjusted difference in difference models, no association was found between PDMP status and opioid-related crime rates or the identification of potential opioid dealers [55]. As for diversion, an interrupted time series study from 2009 to 2012 found significant reductions in rates of diversion of oxycodone, methadone, and morphine in over time in Florida post- PDMP implementation [26]. Finally, no significant trends were identified for other measured drugs in this study (fentanyl, hydrocodone, hydromorphone, buprenorphine, and tramadol). See Additional file 2: Table S4 for individual study details.

### Risk of Bias assessment

A detailed description of risk of bias assessment across the six QUIPs domains by study and overall can be found in Table 2. Overall, study quality was good; a low risk of bias rating was given for 81.8% of studies on study participation, 100.0% on study attrition, 45.5% on PDMP measurement, 54.5% on outcome measurement, 68.3% on study confounding, and 81.8% statistical analysis and reporting. Nine of the included studies were not published in peer-reviewed journals (i.e. working papers, theses) [28, 39–41, 43, 44, 51, 53, 55]. While this does not necessarily indicate poor study quality, it does indicate that the results should be interpreted with caution as these studies have not undergone a rigorous peer review.

**Table 2:** Detailed risk of bias and quality assessment using the QUIPs tool

Study	Study participation	Study attrition	PMP measurement	Outcome measurement	Study confounding	Statistical analysis and reporting
Ali 2017 [24]	moderate	low	moderate	moderate	low	low
Birk 2017 [43]	low	low	moderate	low	low	low
Branham 2018 [27]	moderate	low	moderate	low	low	low
Dave 2017 [44]	low	low	moderate	moderate	low	low
Delcher 2015 [50]	low	low	low	low	low	moderate
Kim 2013 [ 51]	low	low	moderate	moderate	low	low
Kinsell 2015 [ 40]	low	low	low	low	low	moderate
Li 2014 [ 52]	low	low	low	low	low	low
Maughan 2015 [ 25]	low	low	low	low	low	low
Mallatt 2017 [ 55]	low	low	low	low	low	low
McLaughlin 2016 [ 28]	moderate	low	moderate	moderate	low	low
Meara 2016 [ 48]	low	low	moderate	moderate	low	low
Meinhofer 2017 [ 53]	moderate	low	low	low	high	low
Nam 2017 [ 45]	low	low	moderate	moderate	low	low
Patrick 2016 [ 47]	low	low	moderate	moderate	moderate	low
Paulozzi 2011 [ 46]	low	low	moderate	moderate	moderate	low
Pauly 2018 [ 49]	low	low	low	low	low	low
Radakrishnan 2015 [ 39]	low	low	moderate	moderate	low	low
Reifler 2012 [ 42]	low	low	low	low	moderate	low
Reisman 2009 [ 19]	low	low	low	low	moderate	moderate
Simeone 2006 [ 41]	low	low	moderate	moderate	high	high
Surratt 2014 [ 26]	low	low	low	low	moderate	low
Overall % low	81.8%	100.0%	45.5%	54.5%	68.2%	81.8%
Overall % moderate	18.2%	0.0%	54.5%	45.5%	22.7%	13.6%
Overall % high	0.0%	0.0%	0.0%	0.0%	9.1%	4.6%

## Discussion

In this systematic review we sought to identify associations between PDMP status and opioid-related consequences and harms. Twenty-two publications from 12 unique data sets were analysed. Overall, we did not find evidence to indicate that PDMPs were effective in reducing several types of population-level consequences and harms including illicit opioid use, opioid dependence, ED visits, or inpatient discharges. There were, however, very few studies that measured each of these outcomes.

In individual studies, rates of fatal and non-fatal overdoses were higher at baseline in PDMP states, but reductions were observed after PDMP implementation; however, the relationship overall was less clear. Conflicting evidence was found for the association between treatment admissions and PDMP status, with some studies indicating an increase in admissions, and others finding a decrease. An increase in treatment admissions is not necessarily a poor outcome and could be indicative of more people seeking treatment (rather than more people using opioids) due to intervention from PDMP findings, or other arms of opioid related intervention strategies.

While there were no observed effects for the association of PDMPs with harms and consequences related to opioids, PDMPs, if properly operationalized, can be an important piece of a broader opioid strategy. They may work in tandem with other arms of an opioid strategy, rather than functioning as standalone programs. Many studies did not control for the presence and timing of other interventions in their statistical models, which may have masked estimation of the true effect of PDMPs on opioid-related harms. Equally important, in order for PDMPs to function optimally, healthcare providers must use the data whenever they are prescribing an opioid [56]. A recent evidence synthesis by our team found that only 57% of healthcare providers had ever used PDMP data to inform their prescribing decisions (using data from 26 studies), and fewer than 1 in 5 used a PDMP with each prescription. Interventions aimed at increasing PDMP utilization among healthcare providers would impact opioid-related harms and outcomes over time. None of the included studies considered PDMP utilization by healthcare providers when estimating the effect of PDMPs on outcomes.

While only two studies received a rating of high risk of bias on any domain, there were some areas of concern including study confounding, PDMP implementation, and outcome measurement. A study was rated high risk of bias on the study confounding domain if there was no evidence of accounting for important confounders (including demographic trends, other opioid-related interventions in the jurisdiction, time trends, and features of PDMPs) in the study design or statistical models. Concerns with bias on PDMP/outcome measurement mainly stemmed from the timing of measurement. Measuring exposure or outcome only on an annual basis raised concerns about potential misclassification of PDMP status for outcomes during that year (i.e. a prescription could have been dispensed before a PDMP was implemented, but still marked as occurring during a year where the state had a PDMP). Studies that accounted for PDMP status more frequently (i.e. monthly or quarterly) raised less concerns about misclassification. As the body of evidence evolves, a systematic review should be performed focusing on features of PDMPs, such as mandatory use, and potential relationships with opioid-related harms and consequences. More primary studies are required for certain outcomes of interest, including hospital visits, crime and illicit opioid use.

The last year of data covered by the studies captured in this review was 2014. We need more recent and robust data as the opioid crisis has drastically evolved since then, with more recent focus on the very potent fentanyl. Finally, all studies included in this review were conducted in the United States. Future research should seek to determine the impact of PDMPs on opioid-related consequences and harms in other countries.

### Limitations and strengths of the study

This was a rigorously conducted systematic review that synthesized all studies related to the effectiveness of PDMP status in reducing opioid-related harms and consequences. A thorough evaluation of the literature was executed, and the quality of each included study was reviewed to identify any potential biases. We also considered a broad range of patient safety outcomes such as overdose and hospital admissions.

In terms of limitations, we were unable to perform meta-analyses due to heterogeneity across studies and outcomes. Included studies varied in how they measured associations and employed different units of analysis (i.e. person years, state years, states, etc.), populations (i.e. general, insured, treatment, etc.), covariate adjustments, and most importantly, analytic approach. Many studies included in this review used data from TEDS. States contributing data to TEDS can collect either publically- or privately-funded admissions [57]. This variability limits TEDS in its ability to assess admissions outcomes. Additionally, our search was completed in early 2018 and new studies may have been completed. To address this, a single database scan (Ovid MEDLINE) of the literature for 2018 and 2019 was completed by two reviewers (MW and MA) and identified only one potentially relevant study [58]. This well-designed study provides support for the effectiveness of PDMPs, finding that jurisdictions with online PDMPs observed significant reductions in rates of opioid-related hospitalizations.

The objective of this systematic review was to examine the effect of PDMP implementation (initial and over time) on opioid-related harms and consequences. As such, we did not explore outcomes related to other monitored drugs such as benzodiazepines, which may also be affected by PDMP implementation. Furthermore, although some studies accounted for PDMP features, this review did not focus on differences across types of PDMPs or on the effect of legislative changes to PDMP characteristics in regions with pre-existing PDMPs (e.g. mandatory utilization) given the small number of studies for most outcomes of interest.

## Conclusions

Although we did not find evidence to strongly support the overall effectiveness of PDMPs in reducing opioid-related consequences and harms, if operationalized appropriately, they remain a valuable piece of a broader strategy to combat the opioid crisis. The mere presence of PDMPs is a reminder to physicians that they need to be careful when prescribing opioids. PDMPs may not necessarily address the root causes of addiction or guide patients directly to treatment options; however, they can be an important tool for minimizing potential harm and should work in tandem with other opioid preventation programs.

## Additional files

**Additional file 1.** Search Strategy.

**Additional file 2: Table S1.** Illicit and problematic opioid use. **Table S2.** Opioid-related care outcomes. **Table S3.** Opioid-related adverse events. **Table S4.** Opioid-related legal and crime outcomes.

## Data Availability

Not applicable.

## Abbreviations

CADTH: Canadian Agency for Drugs and Technologies in Health
CDC WONDER: Centers for Disease Control and prevention Wide-ranging ONline Data for Epidemiologic Research
CIHI: Canadian Institute for Health Information
CINAHL: Cumulative Index to Nursing & Allied Health Literature
CMA: Canadian Medical Association
ED: Emergency Department
MeSH: Medical Subject Headings
NVSS: National Vital Statistics System
OUD: Opioid Use Disorder
PDMP: Prescription Drug Monitoring Program
PRISMA: Preferred Reporting Items for Systematic reviews and Meta-Analyses
QUIPS: QUality In Prognosis Studies
RADARS: Researched Abuse Diversion and Addiction-Related Surveillance system
RCT: Randomized Controlled Trial
SEDD: State Emergency Department Databases
SID: State Inpatient Databases
TEDS: Treatment Episode Data Set
