# Supplementary material for: The effectiveness of prescription drug monitoring programs at reducing opioid-related harms and consequences: a systematic review
Source: BMC Health Serv Res. 2019 Nov 1;19:784. doi: 10.1186/s12913-019-4642-8 (PMC6825333; doi:10.1186/s12913-019-4642-8)
Supplement: Supplementary file 1 — Additional file 1. Search Strategy. [file 12913_2019_4642_MOESM1_ESM.docx]

**Additional file 1: Search Strategy**


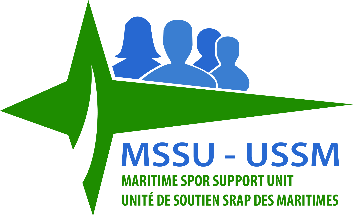


Search Summary:
Effect of prescription drug monitoring programs on opioid-related harms

January 22, 2018

**Searcher:** **Requestor:**

Leah Boulos Maria Wilson (for Mark Asbridge)
[LeahM.Boulos@nshealth.ca](mailto:LeahM.Boulos@nshealth.ca) [Maria.Wilson@dal.ca](mailto:Maria.Wilson@dal.ca)

**Research question:** Do prescription drug monitoring programs reduce opioid-related harms?

**Databases searched/resources consulted:** MEDLINE, Embase, CINAHL with Full Text, PsycINFO, Web of Science

**Target articles:** none provided

# Results Summary

All searches run January 22, 2018.

| ***Database*** | ***Interface*** | ***Dates*** | ***Results*** |
| --- | --- | --- | --- |
| MEDLINE – *Ovid MEDLINE(R) Epub Ahead of Print, In-Process & Other Non-Indexed Citations, Ovid MEDLINE(R) Daily and Ovid MEDLINE(R)* | Ovid | Inception-present | 628 |
| Embase | Embase.com | Inception-present | 1004 |
| CINAHL with Full Text | EBSCOhost | Inception-present | 515 |
| PsycINFO | EBSOChost | Inception-present | 491 |
| Web of Science | Clarivate | Inception-present | 611 |
| **Total** | | | **3249** |
| **Duplicates removed** | | | **1474** |
| **De-duplicated total** | | | **1775** |

# Search Strategies

## MEDLINE

| 1 | Prescription Drug Monitoring Programs/ |
| --- | --- |
| 2 | controlled substance monitoring.tw. |
| 3 | (drug monitoring adj (program* or system* or network*)).tw. |
| 4 | narcotic* monitoring.tw. |
| 5 | prescription drug monitoring.tw. |
| 6 | prescription monitoring.tw. |
| 7 | prescription network*.tw. |
| 8 | or/1-7 |

## Embase

| 1 | 'controlled substance monitoring' |
| --- | --- |
| 2 | 'drug monitoring' NEAR/1 (program* OR system* OR network*) |
| 3 | 'narcotic* monitoring' |
| 4 | 'prescription drug monitoring' |
| 5 | prescription monitoring' |
| 6 | 'prescription network*' |
| 7 | #1 OR #2 OR #3 OR #4 OR #5 OR #6 |

## CINAHL & PsycINFO (identical searches)

| 1 | controlled substance monitoring |
| --- | --- |
| 2 | drug monitoring N1 (program* or system* or network*) |
| 3 | narcotic* monitoring |
| 4 | prescription drug monitoring |
| 5 | prescription monitoring |
| 6 | prescription network* |
| 7 | S1 OR S2 OR S3 OR S4 OR S5 OR S6 |

## Web of Science

| 1 | TS="controlled substance monitoring" |
| --- | --- |
| 2 | TS=("drug monitoring" NEAR/1 (program* or system* or network*)) |
| 3 | TS="narcotic* monitoring" |
| 4 | TS="prescription drug monitoring" |
| 5 | TS="prescription monitoring" |
| 6 | TS="prescription network*" |
| 7 | #6 OR #5 OR #4 OR #3 OR #2 OR #1 |
