# Supplementary material for: The effectiveness of prescription drug monitoring programs at reducing opioid-related harms and consequences: a systematic review
Source: BMC Health Serv Res. 2019 Nov 1;19:784. doi: 10.1186/s12913-019-4642-8 (PMC6825333; doi:10.1186/s12913-019-4642-8)
Supplement: Supplementary file 2 — Additional file 2: Table S1. Illicit and problematic opioid use. Table S2. Opioid-related care outcomes. Table S3. Opioid-related adverse events. Table S4. Opioid-related legal and crime outcomes. [file 12913_2019_4642_MOESM2_ESM.docx]

**Additional file 2: Supplementary Tables**

**Supplementary Table 1. Illicit and problematic opioid use.**

| **Study** | **Outcome** | **N** | **Effect type^1^** | **Effect** | **Lower CI or SE** | **Upper CI** | **p-value** | **Adjusted Y/N** |
| --- | --- | --- | --- | --- | --- | --- | --- | --- |
| **Heroin use** | | | | | | | | |
| **Ali^24^** | heroin use (past year) among all respondents | 507,000 | OR & 95% CI | 1.02 | 0.691 | 1.507 | >0.10 | Y |
| **Ali^24^** | new onset use (past year initiation) among all respondents who don’t already use | 460,000 | OR & 95% CI | 1.228 | 0.616 | 2.447 | >0.10 | Y |
| **McLaughlin^28^** | new onset use (past year) among those who ever used NMPO (similar result for past year & high risk users) | 85,000 | B & SE | -3.76*10^-7^ | 0.00149 | -- | >0.10 | Y |
| **Opioid dependence** | | | | | | | | |
| **Ali^24^** | meets DSM-IV abuse or dependence criteria in past year | 507,000 | OR & 95% CI | 1.03 | 0.814 | 1.304 | >0.10 | Y |

^1^Effect type abbreviations: OR = odds ratio, CI = confidence interval, SE = standard error, B = beta coefficient, IRR = incident rate ratio, GEE = generalized estimating equation coefficient.

**Supplementary Table 2. Opioid-related care outcomes.**

| **Study** | **Outcome** | **N** | **Effect type^1^** | **Effect** | **Lower CI or SE** | **Upper CI** | **p-value** | **Adjusted Y/N** |
| --- | --- | --- | --- | --- | --- | --- | --- | --- |
| **Inpatient discharges** | | | | | | | | |
| **Kinsell^40^** | inpatient discharges related to prescription opioids per 100,000 pop | 96 state months (48 for Florida specifically) | B & SE | 0.135 | 0.091 | -- | 0.138 | Y |
| **Kinsell^40^*** | inpatient discharges related to heroin per 100,000 pop | 96 state months | B & SE | 0.014 | 0.007 | -- | 0.031 | Y |
| **ED visits** | | | | | | | | |
| **Kinsell ^40^** | ED visits for prescription opioids | 96 state months | B & SE | 0.076 | 0.077 | -- | 0.328 | Y |
| **Kinsell^40^*** | ED visits for heroin | 96 state months | B & SE | -0.022 | 0.019 | -- | 0.242 | Y |
| **Maughan^25^** | mean difference in quarterly visit rate per 100,000 population ED visits for prescription opioids | NR | B & 95% CI | 0.8 | -3.7 | 5.2 | 0.74 | Y |
| **Maughan^25^** | mean difference in quarterly visit rate per 100,000 population ED visits for prescription opioids specifically for misuse or abuse | NR | B & 95% CI | 0.8 | -1.9 | 3.4 | 0.57 | Y |
| **Maughan^25^** | mean difference in quarterly visit rate per 100,000 population ED visits for Schedule II opioids | NR | B & 95% CI | -1.8 | -3.9 | 0.3 | 0.09 | Y |
| **Maughan^25^** | mean difference in quarterly visit rate per 100,000 population ED visits for Schedule II opioids specifically for misuse or abuse | NR | B & 95% CI | -1.1 | -2.9 | 0.8 | 0.29 | Y |
| **Treatment admissions** | | | | | | | | |
| **Birk^43^** | all treatment admissions per state year for prescription opioids | 734 state years | B & SE | -0.014 | 0.08 | -- | >0.10 | Y |
| **Branham^27^*** | change in treatment admissions for heroin | NR | B & SE | 0.66 | 0.41 | 0.13 | >0.05 but  excluding Connecticut and Minnesota as outliers finds an increase in heroin admissions | Y |
| **Dave^44^** | change in treatment admissions for prescription opioids | 612 state years | B&SE age-specific models only report | 12-17: -0.400  18-24:  -3.901  25-44:  -1.630  >=45:  -0.217 | 0.231  2.577  1.777  0.159 | -- | p for 12-17 is 0.05<p<0.10  all other are p>0.10 | Y |
| **Dave^44^*** | change in treatment admissions for heroin | 612 state years | B&SE age-specific models only report | 12-17:  -0.013  18-24:  0.588  25-44:  1.720  >=45:  -0.064 | 0.143  2.003  1.401  0.322 | -- | all p>0.10 | Y |
| **Radakrishnan^39^** | treatment admissions for all opioids | 929 state years | B & SE | baseline -0.140  year interaction  -0.037 | 0.066  0.026 | -- | p<0.05  >0.10 | Y |
| **Radakrishnan^39^*** | treatment admissions for heroin | 929 state years | B & SE | -0.030  0.021 | 0.091  0.038 | -- | p>0.10 | Y |
| **Reifler^42^** | treatment admissions for Fentanyl, hydromorphone, methadone, morphine, oxycodone only | NR | B & SE | -0.0218 | 0.0115 | -- | 0.058 | Y |
| **Reisman^19^** | odds a patient entering treatment was abusing prescription opioids in PMP state vs no PMP | NR | OR & 95% CI | 0.775 | 0.764 | 0.785 | <0.05 | Y |
| **Simeone^41^** | change in rate of treatment admissions for Rx O per 100,000 population | NR | rate in PMP & non PMP states in 1997 & 2003 | 2 more per 100,000 in 1997 in PMP states  2 more per 100,000 in 2003 in PMP states | -- | -- | NR | N |

* means heroin only. ^1^Effect type abbreviations: OR = odds ratio, CI = confidence interval, SE = standard error, B = beta coefficient, IRR = incident rate ratio, GEE = generalized estimating equation coefficient.

**Supplementary Table 3. Opioid-related adverse events.**

| **Study** | **Outcome** | **N** | **Effect type^1^** | **Effect** | **Lower CI or SE** | **Upper CI** | **p-value** | **Adjusted Y/N** |
| --- | --- | --- | --- | --- | --- | --- | --- | --- |
| **Fatal overdoses** | | | | | | | | |
| **Birk^43^** | Change in opioid-related deaths | 700 state years | B & SE | -0.056 | 0.10 | -- | >0.10 | Y |
| **Delcher^50^** | Oxycodone-caused mortality (in lethal concentrations) | 120 months, 7804 deaths for oxycodone | B & SE | -24.761 | 9.3146 | -- | 0.0079 | Y |
| **Delcher^50^** | Non-oxycodone opioid overdoses | 120 months | B | 2.77 | -- | -- | 0.7265 | Y |
| **Delcher^50^*** | Heroin overdoses | 120 months | B | 0.50 | -- | -- | 0.8608 | Y |
| **Kim^51^** | Opioid-related poisoning deaths | 510 state years | IRR 95% CI | 0.97 | 0.89 | 1.06 | 0.55 | Y |
| **Kinsell^40^** | All prescription opioid deaths | 96 state months | B & SE | 0.002 | 0.071 | -- | 0.974 | Y |
| **Kinsell^40^*** | Heroin deaths | 96 state months | B & SE | -0.009 | 0.009 | -- | 0.296 | Y |
| **Li (overall)^52^** | Drug overdose mortality | Results also remained consistent when opioid overdose mortality was used as the outcome | RR & 95% CI | 1.11 | 1.02 | 1.21 | <0.05 | Y |
| **Meinhofer^53^** | Prescription opioid deaths | 2800 state-year-quarter | B & SE | -0.02 | 0.08 | -- | >0.10 | Y |
| **Meinhofer^53^*** | Heroin deaths | 2800 state-year-quarter | B & SE | -0.03 | 0.09 | -- | >0.10 | Y |
| **Nam^45^** | Fatal prescription opioid overdoses | NR | B & 95% CI | 0.02 | -0.81 | 0.84 | 0.97 | Y |
| **Nam^45^** | All opioids fatal overdose | NR | B & 95% CI | -0.11 | -0.57 | 0.34 | 0.62 | Y |
| **Nam^45^** | Methadone fatal overdose | NR | B & 95% CI | 0.17 | -0.47 | 0.82 | 0.59 | Y |
| **Patrick^47^** | Prescription opioid fatal overdose per 100,000 pop at baseline | 34 states | B & 95% CI | -1.12 | -1.68 | -0.55 | <0.001 | Y |
| **Patrick^47^** | Prescription opioid fatal overdose per 100,000 pop over time | 34 states | B & 95% CI | 0.11 | 0.00 | 0.23 | 0.06 | Y |
| **Patrick^47^** | Prescription opioid + heroin + opium fatal overdose per 100,000 pop at baseline | 34 states | B & 95% CI | -1.19 | -1.80 | -0.58 | 0.001 | Y |
| **Patrick^47^** | Prescription opioid + heroin + opium fatal overdose per 100,000 pop over time | 34 states | B & 95% CI | 0.18 | 0.05 | 0.30 | 0.006 | Y |
| **Paulozzi^46^** | prescription opioid overdose mortality rate per 100,000 person years (over time) | 357 state years | B | 0.09 | -- | -- | 0.3437 | Y |
| **Radakrishnan^39^** | log opioid overdose deaths | 612 state years | B & SE | baseline: 0.050  pmp*yr: 0.040 | 0.096  0.025 | -- | >0.10  >0.10 | Y |
| **Radakrishnan^39^*** | log heroin overdose deaths | 612 state years | B & SE | baseline: -0.026  pmp*yr: 0.061 | 0.155  0.047 |  | >0.10  >0.10 | Y |
| **Non-fatal overdose** | | | | | | | | |
| **Meara^48^** | Proportion of beneficiaries with non-fatal prescription opioid overdose | 8,693,212  person years | B & SE | -0.018 | 0.021 | -- | >0.10 | Y |
| **Pauly^49^** | Rate of change of prescription opioid poisonings over time | 6600 state months | GEE & 95% CI | -0.005 | -0.008 | -0.003 | <0.0001 over time, decreased more quickly over time in PMP states | Y |
| **Intentional poisonings** | | | | | | | | |
| **Reifler^42^** | intentional opioid poisonings Fentanyl, hydromor-phone, methadone, morphine, oxycodone only over time in PMP vs non PMP states as baseline | NR | B & SE | 0.3292 | 0.1647 | -- | 0.046 | Y |

* means heroin only. ^1^Effect type abbreviations: OR = odds ratio, CI = confidence interval, SE = standard error, B = beta coefficient, IRR = incident rate ratio, GEE = generalized estimating equation coefficient.

**Supplementary Table 4. Opioid-related legal and crime outcomes.**

| **Study** | **Outcome** | **N** | **Effect type^1^** | **Effect** | **Lower CI or SE** | **Upper CI** | **p-value** | **Adjusted Y/N** |
| --- | --- | --- | --- | --- | --- | --- | --- | --- |
| **Crime rates** | | | | | | | | |
| **Mallatt^55^** | DID for opioid-related crimes per capita | 24384 county-months | B & SE | -0.162 | 0.0956 | -- | 0.243 | Y |
| **Mallatt^55^*** | DID for heroin-related crimes per capita | 24780  county-months | B & SE | 0.239 | 0.288 | -- | 0.654 | Y |
| **Potential dealers identified** | | | | | | | | |
| **Mallatt^55^** | DID for possible opioid dealers identified per capita | 24780  county-months | B & SE | -0.0174 | 0.0257 | -- | 0.246 | Y |
| **Mallatt^55^*** | DID for possible heroin dealers identified per capita | 24780  county-months | B & SE | 0.112 | 0.059 | -- | 0.058 | Y |
| **Diversion** | | | | | | | | |
| **Surratt^26^** | Change in population (per 100,000 for all) diversion rates of oxycodone over time in PMP vs non PMP states | 15 state quarters/219 counties | B & SE | -1.31 | 0.60 | -- | 0.01<p<0.05 | N |
| **Surratt^26^** | Change in population diversion rates of fentanyl over time in PMP vs non PMP states | 15 state quarters/219 counties | B & SE | -0.02 | 0.02 | -- | >0.10 | N |
| **Surratt^26^** | Change in population diversion rates of hydrocodone over time in PMP vs non PMP states | 15 state quarters/219 counties | B & SE | -0.49 | 0.28 | -- | 0.05<p<0.10 | N |
| **Surratt^26^** | Change in population diversion rates of hydromorphone over time in PMP vs non PMP states | 15 state quarters/219 counties | B & SE | 0.01 | 0.05 | -- | >0.10 | N |
| **Surratt^26^** | Change in population diversion rates of morphine over time in PMP vs non PMP states | 15 state quarters/219 counties | B & SE | -0.13 | 0.06 | -- | <0.01<p<0.05 | N |
| **Surratt^26^** | Change in population diversion rates of methadone over time in PMP vs non PMP states | 15 state quarters/219 counties | B & SE | -0.23 | 0.07 | -- | 0.01<p<0.001 | N |
| **Surratt^26^** | Change in population diversion rates of buprenorphine over time in PMP vs non PMP states | 15 state quarters/219 counties | B & SE | -0.05 | 0.04 | -- | >0.10 | N |
| **Surratt^26^** | Change in population diversion rates of tramadol over time in PMP vs non PMP states | 15 state quarters/219 counties | B & SE | -0.04 | 0.04 | -- | >0.10 | N |

* means heroin only. ^1^Effect type abbreviations: OR = odds ratio, CI = confidence interval, SE = standard error, B = beta coefficient, IRR = incident rate ratio, GEE = generalized estimating equation coefficient.
